# Catching Up on Mortality, Falling Behind on Disability: A Dynamic Decomposition of the Health-Adjusted Life Expectancy Gap between Higher-Income and Lower-Income Countries

**DOI:** 10.5334/aogh.5458

**Published:** 2026-08-28

**Authors:** Xue-Qi Li, Wei Hu, Bo Yan, Jie Sun, Jun-Yan Xi

**Affiliations:** 1 Chengdu Integrated TCM and Western Medicine Hospital (Chengdu First People’s Hospital), Chengdu 610041, Sichuan, China; 2 School of Public Administration, Sichuan University, Chengdu 610065, Sichuan, China; 3 Department of Epidemiology, School of Public Health, Sun Yat-sen University, Guangzhou 510080, Guangdong, China; 4 Guangzhou Medical University Library, Guangzhou 511436, Guangdong, China; 5 Institute for the Control of Infectious Diseases, Guizhou Center for Disease Control and Prevention, Guiyang 550004, Guizhou, China; 6 Department of Medical Statistics, School of Public Health, Sun Yat-sen University, Guangzhou 510080, Guangdong, China; 7 Sun Yat-sen Global Health Institute, Sun Yat-sen University, Guangzhou 510080, Guangdong, China; 8 Center for Health Information Research, Sun Yat-sen University, Guangzhou 510080, Guangdong, China

**Keywords:** decomposition analysis, disability, health-adjusted life expectancy, health inequality, mortality

## Abstract

*Background:* Global health inequalities are conventionally assessed through the mortality gap, yet the joint contribution of mortality and disability to health-adjusted life expectancy (HALE) gap and their dynamic evolution remain poorly understood. We aim to decompose the HALE gap between higher-income and lower-income countries into mortality and disability drivers, separate long-term trends from transient fluctuations, and reveal heterogeneity within the low-income group.

*Methods:* Using data from the Global Burden of Disease Study for 199 countries (122 higher-income and 77 lower-income) from 2010 to 2019, we decomposed the HALE gap by age and cause via Horiuchi’s method. Empirical mode decomposition separated each contribution time series into a long-term trend and residual fluctuations, enabling classification into dynamic archetypes: mortality catch-up, mortality divergence, disability catch-up, or disability divergence. Principal component analysis and partitioning around medoids clustering explored heterogeneity among lower-income countries, and generalized additive models assessed variation along the Socio-demographic Index (SDI) gradient.

*Results:* The HALE gap narrowed from 9.10 to 7.93 years, with mortality contributing 6.87 years and disability 1.06 years in 2019. Mortality catch-up was concentrated in childhood infections and maternal conditions, while mortality divergence occurred for non-communicable diseases in adults. Disability divergence was driven by mental, musculoskeletal, and sense organ disorders. Low-income countries clustered into a high-mortality group (mainly sub-Saharan Africa and South Asia) and a disability-emerging group (Asia, Latin America). Along the development gradient, disability progressively dominated the HALE gap.

*Conclusions:* The converging HALE gap masks a diverging disability gap. Sustainable progress in global health requires explicitly targeting the growing burden of non-fatal conditions alongside persistent mortality inequalities. The dynamic framework introduced here offers a reusable monitoring tool for health inequality.

## Introduction

Despite decades of gains in life expectancy worldwide, the health inequality between high-income and low-income countries has not narrowed uniformly [1–3]. Increasing evidence suggests that longer lives do not necessarily translate into healthier lives. In low-income settings, years lived with disability may be expanding. This makes inequalities in health-adjusted life expectancy (HALE) far more complex than mortality differences alone [4–6]. Yet a fundamental question remains unanswered: Is the remaining health inequality driven primarily by deaths that could still be prevented, or by disability that continues to be insufficiently addressed? [7] And beneath the aggregate trend, do low-income countries share a common pattern of disadvantage, or does each face a distinct set of health challenges? [8]

Although prior work has recognized the importance of disability and has compared health inequality across individual countries, existing analytical frameworks remain limited in three crucial respects. First, they capture the health inequality at a single point in time, offering a static snapshot that cannot reveal whether the underlying drivers are structurally converging or diverging over the long run [2, 3]. Second, even when disability is considered, it is rarely decomposed jointly with mortality in a way that allows their contributions to be tracked as co-evolving, frequency-specific trajectories [9, 10]. This distinction is crucial for separating persistent trends from transient fluctuations. Third, cross-country comparisons, where they exist, are typically framed as isolated case studies or simple rankings rather than as a systematic, data-driven classification of the distinct structural patterns that differentiate one low-income country from another [11, 12]. In short, what has been missing is a dynamic, frequency-aware decomposition that treats mortality and disability as parallel, interacting streams and that simultaneously uncovers the heterogeneous disease-burden profiles within the low-income world [13].

Here, we introduce such a framework. Our approach integrates age–cause decomposition with frequency-domain trend separation via empirical mode decomposition. For the first time, it jointly quantifies the mortality and disability drivers of the HALE gap as they evolve. We map the dynamic structure of the HALE gap between high-income and low-income countries, identify the key age–cause combinations that systematically widen or narrow the gap, reveal heterogeneous trajectories within the low-income group, and characterize how the HALE gap transforms along the socio-demographic development gradient. This dynamic monitoring framework is intended to provide fine-grained, actionable targets for global health governance.

## Methods and Materials

### Data sources

We used data from the Global Burden of Disease Study 2021 (GBD), covering the period 2010–2019. For each country, year, sex, and age group, we extracted the number of deaths, years lived with disability (YLD), and population size for 22 level-2 causes. Age was categorized into 19 groups from <1, 1–4, 5–9, …, ≥85 years. The 22 causes included: HIV/AIDS and sexually transmitted infections, respiratory infections and tuberculosis, enteric infections, neglected tropical diseases and malaria, other infectious diseases, maternal and neonatal disorders, nutritional deficiencies, neoplasms, cardiovascular diseases, chronic respiratory diseases, digestive diseases, neurological disorders, mental disorders, substance use disorders, diabetes and kidney diseases, skin and subcutaneous diseases, sense organ diseases, musculoskeletal disorders, other non-communicable diseases, transport injuries, unintentional injuries, and self-harm and interpersonal violence. The annual Socio-demographic Index (SDI) was also obtained from GBD. Detailed descriptions of the GBD methodology and cause hierarchy are published elsewhere [1]. We grouped countries according to the World Bank income classification for the 2023 fiscal year [14]. The “higher-income” group combined countries classified as high-income or upper-middle-income, and the “lower-income” group combined those classified as low-income or lower-middle-income. A total of 199 countries were included, with 122 in the higher-income group and 77 in the lower-income group.

### Statistical analysis

We developed a multi-step decomposition framework to examine the dynamic structure of the HALE gap between higher-income and lower-income countries. The analysis proceeded in five stages. First, for each year, sex, and income group, we calculated age-specific mortality and YLD rates for 22 level-2 causes. We then constructed abridged life tables and applied the Sullivan method to compute HALE at birth [15, 16] **(Appendix Text S1)**. Second, for each year, we decomposed the between-group HALE gap into additive contributions from mortality and disability using numerical decomposition (Horiuchi’s method) [17, 18]. We defined two counterfactual HALE scenarios. In the first, we replaced the mortality rates of the lower-income group with those of the higher-income group, keeping their own disability rates unchanged. In the second, we made the symmetric replacement for disability. The HALE gap attributable to mortality is then the difference between this first counterfactual HALE and the actual HALE of the lower-income group. This procedure yielded a cause–age matrix of contributions for mortality and disability separately each year **(Appendix Text S2)**. Third, to understand how these contributions evolved, we examined the time series of each cause–age contribution over 2010–2019. We used empirical mode decomposition to separate each series into a long-term trend, cyclical fluctuations, and residual noise [19, 20]. Based on the overall direction of the long-term trend, each cause–age combination was classified into a dynamic archetype: mortality catch-up (the mortality contribution declined over time, narrowing the HALE gap), mortality divergence (the mortality contribution increased, widening the HALE gap), disability catch-up (the disability contribution declined), or disability divergence (the disability contribution increased). This classification was applied separately to the mortality and disability matrices, so that each age–cause cell could be assigned a distinct dynamic label for each effect type **(Appendix Text S3)**. Fourth, to explore heterogeneity within the lower-income group, we repeated the decomposition for each lower-income country against the higher-income reference group and extracted each country’s cause–age contribution vectors. We performed principal component analysis and partitioning around medoids clustering to identify distinct structural profiles **(Appendix Text S4)**. Fifth, we examined how the mortality and disability contributions varied along the development gradient by plotting country-year observations for all 199 countries against their SDI values and fitting generalized additive models separately for mortality and disability contributions.

## Results

### HALE gap between higher-income and lower-income countries, 2010–2019

**Figure 1:**
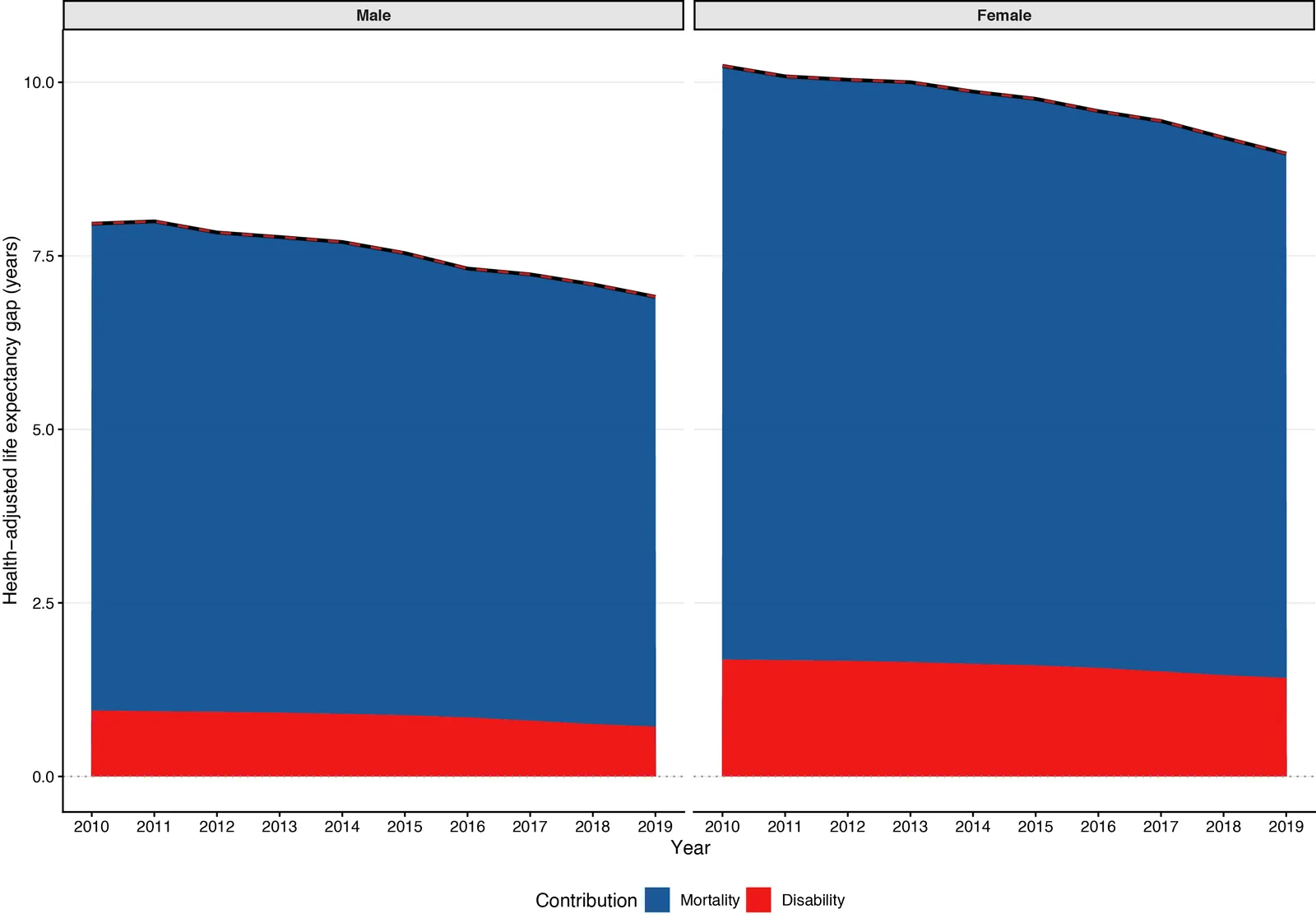
Decomposition of the HALE gap by sex. *Notes:* Stacked areas represent contributions of mortality (blue) and disability (red) to the overall HALE gap. The dashed red line shows the long-term trend extracted by empirical mode decomposition. Panels display results for males (left) and females (right). Positive values indicate higher HALE in higher-income countries.

Between 2010 and 2019, the HALE gap between higher-income and lower-income countries narrowed from 9.10 to 7.93 years for both sexes combined. Mortality differences accounted for the majority of the HALE gap throughout, declining from 7.80 to 6.87 years, while the disability contribution decreased from 1.29 to 1.06 years. The HALE gap was consistently larger for females than for males: the female HALE gap fell from 10.24 to 8.97 years, and the male HALE gap from 7.96 to 6.91 years (Figure 1).

### Age- and cause-specific contributions to the HALE gap in 2019

**Figure 2:**
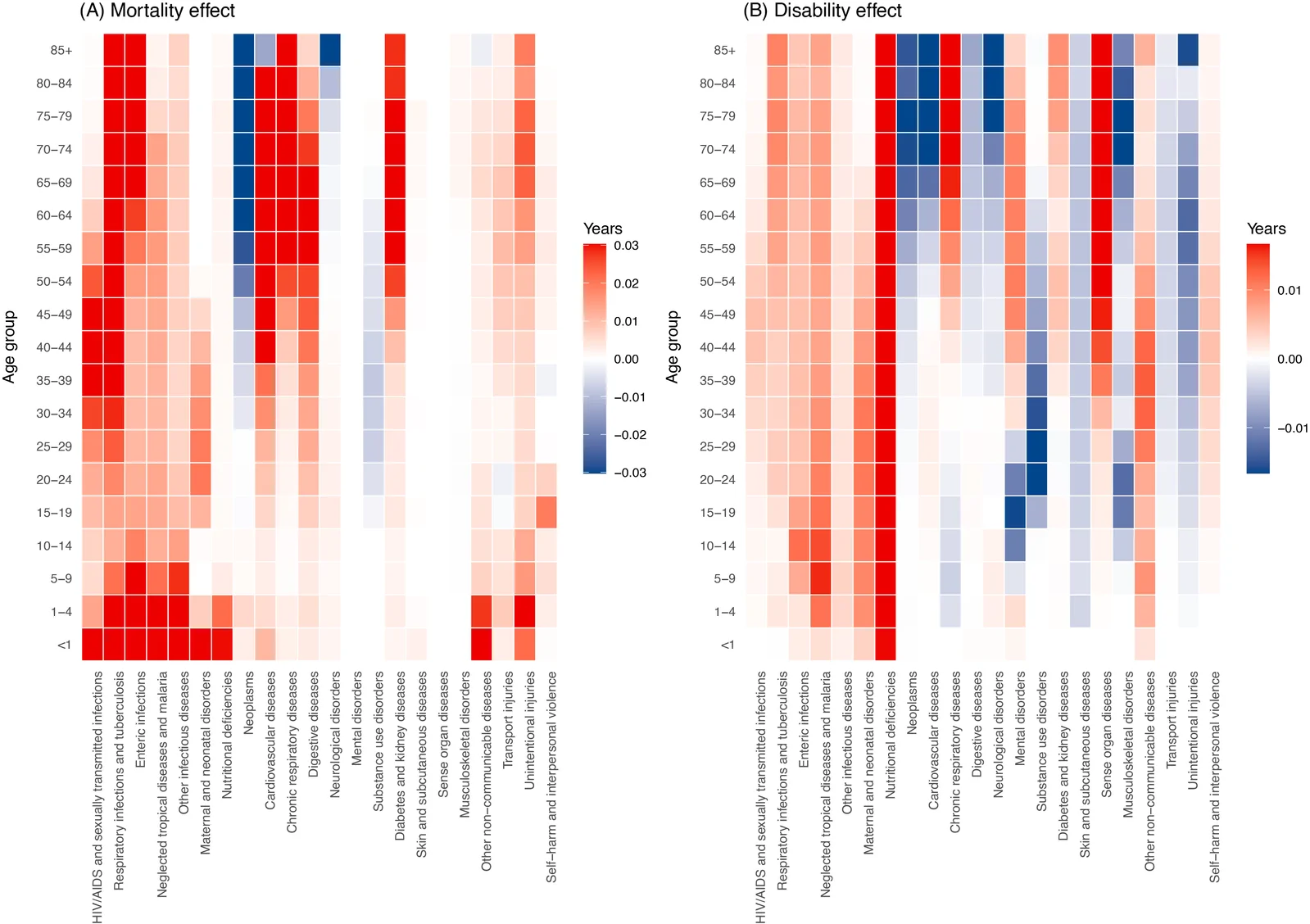
Heatmaps of age–cause contributions to the HALE gap between higher-income and lower-income countries, 2019. *Notes:* Panel **A** (left): mortality effect; Panel **B** (right): disability effect. Red cells represent causes and ages that widen the HALE gap (favoring higher-income countries), while blue cells represent contributions that narrow the HALE gap.

In 2019, the decomposition of the HALE gap by age and cause revealed distinct patterns for mortality and disability contributions (Figure 2). For mortality, the largest widening effects were attributable to maternal and neonatal disorders among children under one year (0.99 years), respiratory infections and tuberculosis (0.32 years), and cardiovascular diseases among adults aged 60–69 years (up to 0.21 years), while neoplasms and neurological disorders at older ages narrowed the HALE gap (e.g., −0.08 years and −0.04 years at 85+ years). For disability, the leading widening contributions came from sense organ diseases in the oldest age groups (e.g., 0.06 years at 85+ years) and nutritional deficiencies in early childhood (e.g., 0.04 years at 1–4 years), whereas neurological disorders and cardiovascular diseases (e.g., −0.05 years and −0.02 years at 85+ years), and substance use disorders (e.g., −0.02 years at 25–29 years) acted to reduce the HALE gap.

### Classification of age–cause dynamic archetypes

**Figure 3:**
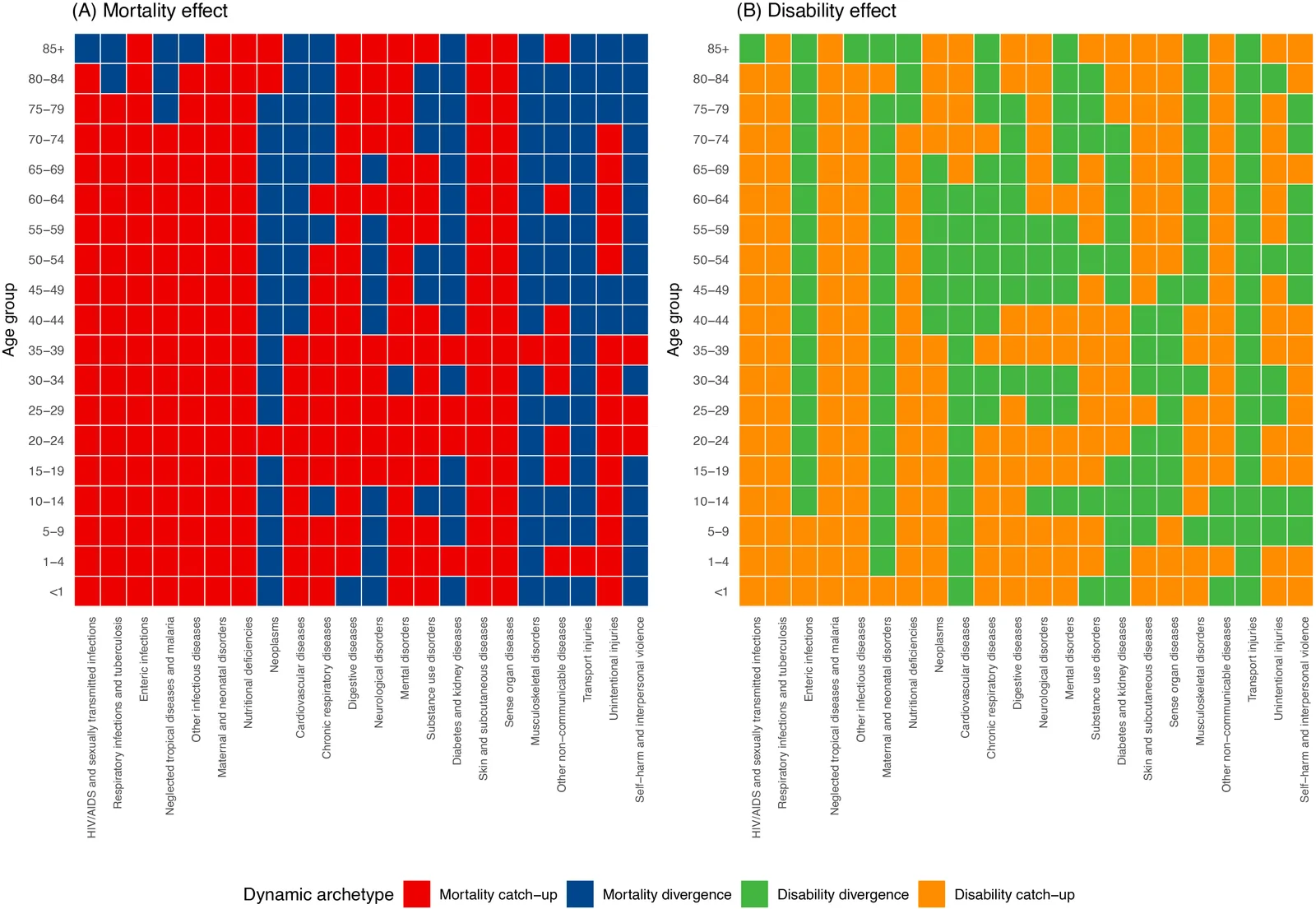
Dynamic archetypes of age–cause contributions over 2010–2019. *Notes:* Panel **A** (left): mortality effect; Panel **B** (right): disability effect. Each cell corresponds to an age–cause combination and is colored according to its trend direction: red, mortality catch-up (trend narrowing the HALE gap); blue, mortality divergence (trend widening the HALE gap); green, disability divergence; orange, disability catch-up.

Among age–cause contributions decomposed by mortality and disability, four dynamic archetypes were identified based on the direction of trend change (mortality catch-up, mortality divergence, disability catch-up, and disability divergence) **(Appendix Figure S1)**. Mortality catch-up was concentrated in infectious diseases (notably respiratory infections and tuberculosis, enteric infections, and HIV/AIDS) and maternal and neonatal disorders, with the largest contributions observed in children under five years. Mortality divergence was driven primarily by non-communicable diseases—particularly cardiovascular diseases, neoplasms, and diabetes—among adults aged 40 years and older. Disability catch-up was observed for certain infectious and nutritional conditions, although its magnitude was generally smaller than that of mortality catch-up. Disability divergence was dominated by mental disorders, musculoskeletal disorders, and sense organ diseases, especially in middle-aged and older adults (Figure 3).

### Heterogeneity in HALE gap structure among lower-income countries

**Figure 4:**
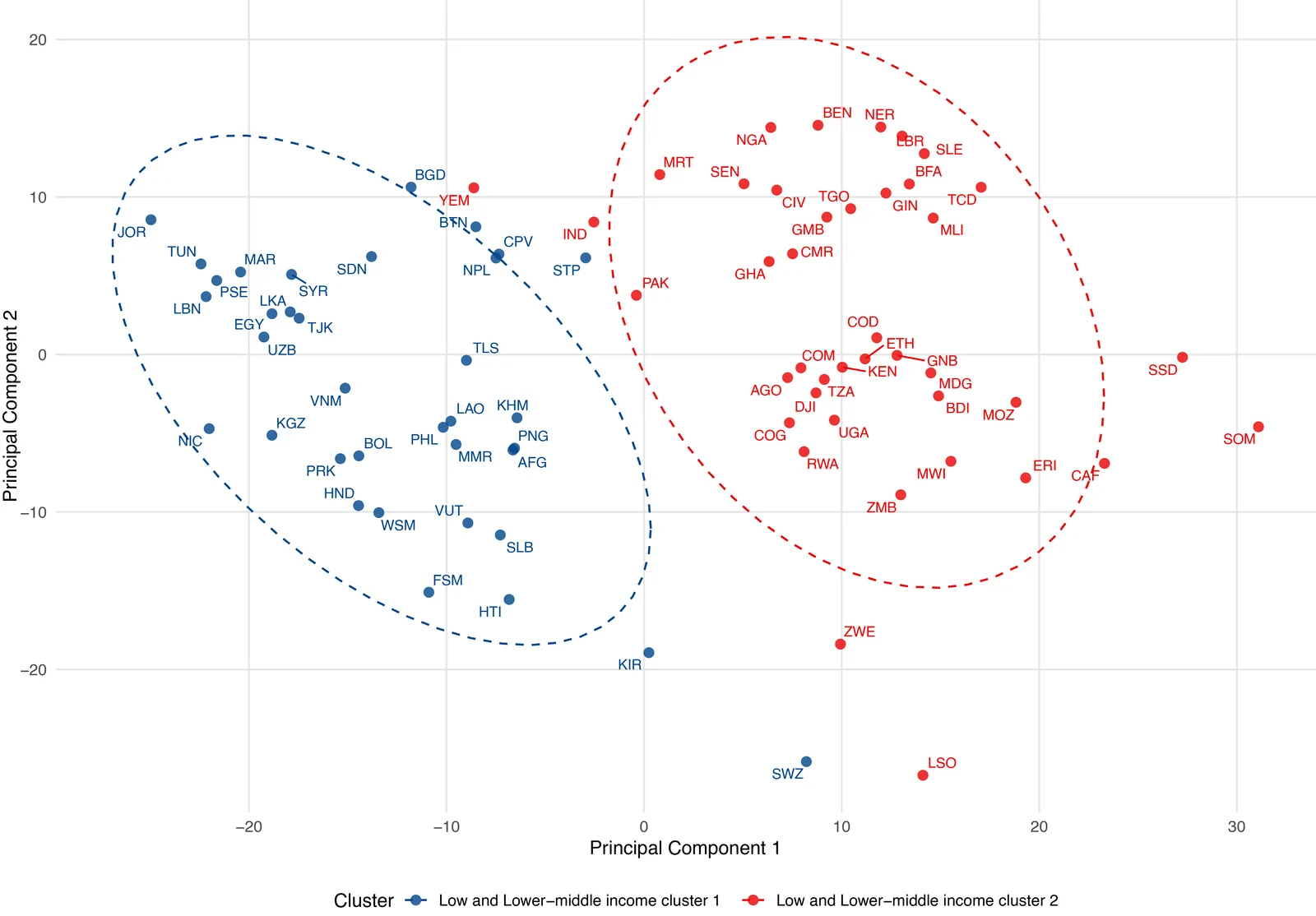
Clustering of lower-income countries by age–cause contribution profiles. *Notes:* Principal component analysis of lower-income countries based on their age–cause contribution vectors. Countries are colored by cluster membership, and ellipses denote 95% confidence regions. Country labels use ISO 3166-1 alpha-3 codes.

Cluster analysis of lower-income countries based on their age–cause contribution profiles identified two distinct patterns (Figure 4). Cluster 1 (36 countries, predominantly in Asia, Latin America, the Middle East, and parts of the Pacific) exhibited a comparatively smaller HALE gap with a lower mortality component and a relatively more prominent disability contribution, particularly from musculoskeletal disorders and sense organ diseases at older ages. Cluster 2 (41 countries, mainly in sub-Saharan Africa and South Asia) showed a substantially larger HALE gap driven overwhelmingly by mortality, with very high contributions from infectious diseases (respiratory infections, enteric infections, HIV/AIDS), maternal and neonatal disorders, and cardiovascular diseases across all ages **(Appendix Figure S2)**. These results revealed marked heterogeneity among lower-income countries, highlighting that one cluster continues to bear a heavy burden of preventable deaths while the other is transitioning toward a pattern in which disability plays a larger relative role in the HALE gap.

### HALE gap drivers along the socio-demographic development gradient

**Figure 5:**
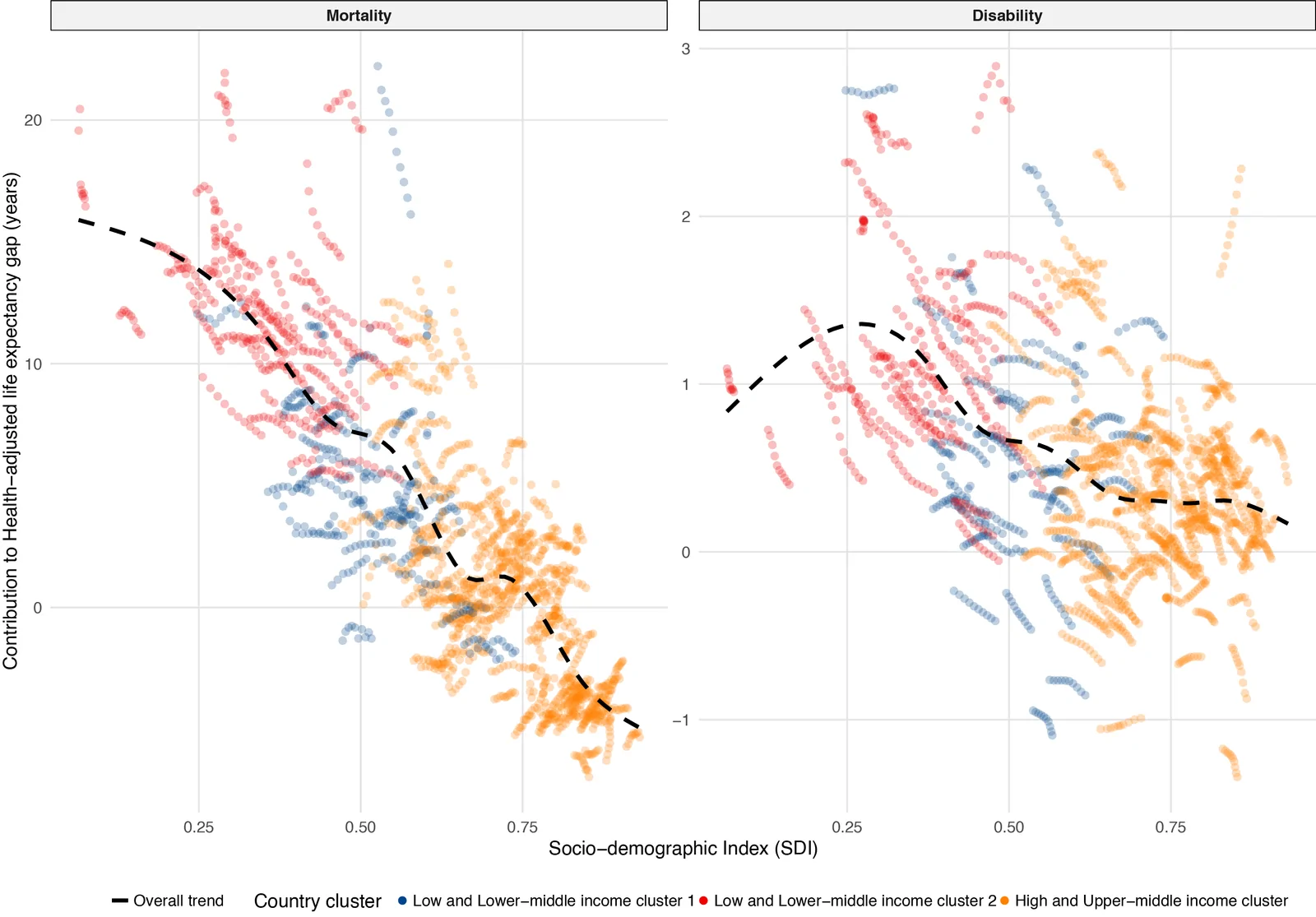
Mortality and disability contributions to the HALE gap as a function of the Socio-demographic Index. *Notes:* Points represent country-year observations, colored by the clusters identified in Figure 4. Solid curves are fitted by generalized additive models, showing the overall trend.

The gradient analysis showed that the HALE gap transitions from a mortality-dominated pattern at lower socio-demographic development levels toward a disability-dominated pattern at higher levels. Among lower-income countries, Cluster 1 (primarily Asian and Latin American) exhibited a declining mortality contribution and a growing disability contribution as SDI increased, with the disability contribution becoming the dominant driver at SDI values above approximately 0.55. Cluster 2 (mainly sub-Saharan African) showed persistently large mortality contributions across the observed SDI range, though a modest shift toward disability was also apparent. Higher-income countries, situated at the upper end of the SDI spectrum (SD > 0.64), displayed an even more pronounced pattern: mortality contributions were considerably smaller, while disability contributions accounted for virtually the entire HALE gap, highlighting the epidemiological transition from deaths to disability as the primary source of cross-national health inequality (Figure 5).

## Discussion

In this study, we introduced a dynamic decomposition framework that integrates age–cause decomposition with frequency-domain trend separation to examine the evolving structure of the HALE gap between higher-income and lower-income countries. Our analysis yielded three overarching findings. First, the overall HALE gap narrowed over the past decade, but this convergence was driven almost entirely by mortality improvements; the disability contribution of the HALE gap persisted and even expanded for several causes. Second, we identified a set of archetypal dynamic patterns—mortality catch-up concentrated among childhood infections, mortality divergence driven by cardiovascular diseases in middle-aged and older adults, and a striking disability divergence for mental and musculoskeletal disorders. Third, lower-income countries did not form a single block: clustering revealed distinct structural profiles ranging from high-mortality, infection-dominated patterns to profiles in which disability already plays a prominent role. These findings provide a new, dynamic surveillance tool for global health inequality and carry direct implications for the allocation of health resources in the post-Sustainable Development Goals era.

### Mortality catch-up coexists with disability divergence

A central contribution of this work is the simultaneous quantification of mortality and disability drivers over time, which reveals a tension that would be invisible to mortality-only decompositions. While deaths from infectious diseases and maternal conditions continued their long-term decline in lower-income countries—contributing to a narrowing of the mortality gap—disability from mental disorders, musculoskeletal conditions, and sense organ diseases steadily increased the disability gap. This pattern aligns with the “compression of morbidity” hypothesis observed in some high-income settings. At the same time, it suggests that lower-income countries may be experiencing an expansion of morbidity even as mortality falls [3, 21, 22]. The implication is clear: if the global health community remains focused solely on averting deaths, the disability contribution of the health inequality will continue to grow, undermining the very notion of “healthy longevity” [2, 23]. Our framework can serve as an early-warning system by highlighting the specific age–cause combinations where disability divergence is accelerating, thereby guiding investment toward mental health, rehabilitation, and chronic pain management. Such areas that have historically received limited attention in global health financing.

### Heterogeneity within the lower-income group challenges one-size-fits-all policies

Another key finding is the substantial structural heterogeneity among lower-income countries. The cluster analysis revealed that some countries still face a health inequality dominated by preventable deaths from infections and maternal conditions, while others have already transitioned to a profile in which non-communicable diseases and disability play a larger role. These distinct profiles are not entirely explained by SDI, suggesting that country-specific factors such as health system capacity, governance, and the availability of rehabilitative services shape the composition of the health inequality beyond what income or education levels would predict [24–26]. This finding cautions against the common practice of treating “lower-income countries” as a single analytic category and highlights the need for tailored, country-specific strategies. The data-driven classification we provide can help multilateral agencies and national governments identify peer countries with similar structural profiles and design interventions that target each cluster’s most relevant age–cause priorities.

For instance, within the high-mortality Cluster 2, countries such as Chad, the Central African Republic, and Somalia exhibited HALE gaps of approximately ten years, driven almost entirely by deaths from infectious diseases and maternal conditions [27]. By contrast, within the disability-emerging Cluster 1, countries including Vietnam, Bolivia, and the Philippines showed gaps of around five years. In these countries, disability from musculoskeletal disorders and mental conditions already accounted for more than 20% of the total gap [28]. These contrasts reveal that the “lower-income” label conceals fundamentally different health realities: a health system in Chad faces markedly different demands from one in Vietnam, and global health financing mechanisms should recognize this diversity rather than imposing uniform eligibility criteria [29]. A small number of countries lay at the boundary between the two clusters, suggesting active epidemiological transition. Rwanda, for example, despite its lower-income status, exhibited a contribution profile closer to Cluster 1, likely reflecting sustained health system strengthening over the past two decades [30, 31]. Such cases illustrate how targeted investment and strong governance can accelerate the epidemiological transition independently of income growth. Taken together, these examples suggest that the eligibility criteria for concessional financing and development assistance should incorporate not only income status but also the structural composition of the disease burden, to avoid withdrawing support prematurely from countries that are transitioning epidemiologically yet still face a substantial burden of non-communicable diseases and disability [32].

### A dynamic surveillance framework for global health inequality

Beyond the substantive findings, this study demonstrated the feasibility and value of treating the health inequality as a dynamic, multidimensional signal rather than a static summary measure. By integrating age–cause decomposition with frequency-domain trend separation, our framework enables the routine monitoring of not only the magnitude of inequality but also its structural composition and temporal dynamics. The separation of long-term trends from short-term fluctuations allowed analysts to distinguish structural divergences that require systemic policy responses from transient variations that may resolve without intervention. As the global health community moves toward the post-2030 agenda, tools that can provide such granular, time-resolved diagnostics will become increasingly important for targeting resources and tracking progress [33–36]. Importantly, the framework is modular and can be adapted to any data source: it can be applied to any setting where comparable age- and cause-specific mortality and disability data are available, including subnational analyses, health system comparisons, or disease-specific tracking. The approach could also be extended to incorporate risk-factor-attributable burdens, economic outcomes, or other dimensions of health inequality, making it a flexible platform for monitoring whether populations are converging not only in length of life but also in the quality of life [37].

### Strengths and limitations

This study has several strengths. It is, to our knowledge, the first to jointly decompose mortality and disability contributions to the HALE gap in a dynamic, frequency-resolved framework that distinguishes long-term trends from short-term fluctuations. The inclusion of all 22 level-2 GBD causes across the full age range provides a comprehensive map of the structural drivers of health inequality. Furthermore, the country-level analysis reveals the heterogeneity masked by income-group aggregates, offering actionable insights for differentiated policy responses.

Several limitations should be acknowledged. First, the analysis relies on GBD estimates, which for many lower-income countries are derived from statistical modeling rather than complete vital registration systems. While measurement error in mortality and YLD rates cannot be ruled out, GBD represents the most rigorously standardized and widely used source of comparable health data across countries, and our focus on the direction and relative magnitude of contributions rather than on their absolute precision reduces the influence of potential biases. Second, YLD estimates may understate the true disability burden in settings with limited diagnostic capacity, potentially attenuating the disability contributions we report. This would, if anything, make our estimates of disability divergence conservative, meaning that the true contribution of disability to the HALE gap may be even larger than we observed. Third, the income-based grouping combines upper-middle-income and high-income countries into a single reference group, which may obscure heterogeneity within the comparator. We chose this grouping because it is policy-relevant and widely used in global health governance. Fourth, our framework is descriptive and does not estimate the causal effects of specific interventions or policies. This is inherent to the design of macro-level decomposition studies, which aim to generate hypotheses and identify priority targets rather than to infer causality. The framework is intended to complement, not replace, causal evaluations of specific programs. Despite these limitations, the proposed approach offers a new lens for monitoring global health inequality and can be updated and refined as data quality and availability improve.

## Conclusion

This study introduced a dynamic, frequency-resolved decomposition framework that simultaneously quantifies the mortality and disability drivers of the HALE gap between higher-income and lower-income countries. Our findings reveal that the observed narrowing of the health inequality over the past decade masks a troubling divergence: while deaths from infectious diseases have been catching up, disability—particularly from mental, musculoskeletal, and sense organ disorders—has been silently widening the divide. Moreover, lower-income countries are not a single block but follow heterogeneous structural trajectories that require tailored, rather than uniform, policy responses. As the global health community transitions from the Sustainable Development Goals to the next era, the persistent and growing contribution of disability demands urgent attention. The framework presented here offered a modular, data-source-agnostic tool for continuously monitoring not only how long people live, but how well they live, and for ensuring that the pursuit of longevity does not leave quality of life behind.

## Data Availability

The full dataset in this study can be obtained from the Institute for Health Metrics and Evaluation (https://www.healthdata.org/). All data generated during this study are included in the manuscript and supporting files.

## Additional File

The additional file for this article can be found as follows:
