# Supplementary material for: Catching Up on Mortality, Falling Behind on Disability: A Dynamic Decomposition of the Health-Adjusted Life Expectancy Gap between Higher-Income and Lower-Income Countries
Source: Ann Glob Health. 2026 Aug 28;92(1):84. doi: 10.5334/aogh.5458 (PMC13523807; doi:10.5334/aogh.5458)
Supplement: Supplementary file 1 [file agh-92-1-5458-s1.pdf]

Catching up on mortality, falling behind on disability: a dynamic decomposition of the health-adjusted life expectancy gap between higher-income and lower-income countries

Appendix

Appendix Text S1. Abridged life table construction and Sullivan's method.................2
Appendix Text S2. Decomposition of the HALE gap 4
Appendix Text S3. Empirical mode decomposition of contribution time series 6
Appendix Text S4. Clustering of lower-income countries..6
Appendix Figure S1. Aggregated long-term trends of the four dynamic archetypes of age-cause contributions to the HALE gap, 2010–2019 7
Appendix Figure S2. Mean age-cause contribution profiles for each cluster of lower-income countries, separately for major mortality and disability effects..............8

Appendix Text S1. Abridged life table construction and Sullivan's method

We constructed abridged life tables using the Chiang method. Let the age intervals be defined as $[x_i, x_i + n_i)$ for $i = 1, \dots, K$, where n_i is the length of the interval, and K is the number of age groups (here $K = 19$). The final interval is open-ended ($n_K = \infty$). For each age group i, we obtained the age-specific death rate ${}_n m_i$ from the ratio of total deaths to person-years lived.

The probability of dying in interval i is

$$ {}_n q_i = \frac{n_i \cdot {}_n m_i}{1 + (n_i - {}_n a_i) \cdot {}_n m_i}, \qquad i = 1, \dots, K-1 $$

and ${}_n q_i = 1$ for the open-ended group. The quantity ${}_n a_i$ is the average number of years lived in the interval by those who die. For the youngest age group (<1 year), we set ${}_n a_1 = 0.1$; for all other closed intervals, we assumed ${}_n a_i = n_i/2$; for the open-ended interval, we used ${}_\infty a_K = 1/{}_n m_K$.

Given the radix $l_1 = 1$, the survival function was computed recursively:

$$ l_{i+1} = l_i(1 - {}_n q_i), \qquad i = 1, \dots, K-1 $$

The number of deaths in each interval is ${}_n d_i = l_i - l_{i+1}$. Person-years lived in interval i is

$$ {}_n L_i = \begin{cases} n_i \cdot l_{i+1} + {}_n a_i \cdot {}_n d_i, & i = 1, \dots, K-1 \\ \dfrac{l_K}{{}_n m_K}, & i = K \end{cases} $$

Total person-years beyond age x_i is $T_i = \sum_{j=i}^{K} L_j$, and life expectancy at the start of interval i is $e_i = T_i / l_i$. Life expectancy at birth corresponds to e_1.

Health-adjusted life expectancy (HALE) was computed using the Sullivan method, which incorporates age-specific disability prevalence into the life table. Let ${}_n \pi_i$ denote the all-cause YLD rate per capita for age interval i. The healthy person-years lived in interval i are computed as

$$ {}_{n}L'_{i} = (1 - {}_{n}\pi_{i}) \cdot {}_{n}L_{i} $$

Total healthy person-years $T'_i = \sum_{j=i}^{K} L'_j$, and HALE at the start of interval i is $HALE_i = T'_i / l_i$.

Appendix Text S2. Decomposition of the HALE gap

Let superscripts H and L denote the higher-income and lower-income groups, respectively. For a given year, the observed HALE gap is

$$\Delta = HALE^{H} - HALE^{L}$$

The HALE of each group is a function of two matrices: the cause-age mortality matrix $\mathbf{M}$ (dimension $K \times C$, where $C = 22$ causes) and the cause-age YLD rate matrix $\mathbf{D}$. Thus, we write $HALE = f(\mathbf{M}, \mathbf{D})$. The function f first sums the cause-specific rates to obtain all-cause mortality and all-cause YLD rates, constructs a life table, and applies the Sullivan method as described in Appendix Text S1.

To isolate the contribution of mortality differences, we construct a counterfactual HALE by hypothetically replacing the mortality rates of the lower-income group with those of the higher-income group, while keeping its own disability rates unchanged. This counterfactual HALE, written as $f(\mathbf{M}^{H}, \mathbf{D}^{L})$, represents the health that the lower-income group would attain if it experienced the mortality schedule of the higher-income group but retained its own disability burden. The mortality contribution to the gap is then

$$\Delta_{\text{mort}} = f(\mathbf{M}^{H}, \mathbf{D}^{L}) - f(\mathbf{M}^{L}, \mathbf{D}^{L})$$

A negative value indicates that mortality rates in the higher-income group are lower (i.e., they narrow the gap). Analogously, to quantify the contribution of disability differences, we start from the mortality rates of the higher-income group and build a second counterfactual HALE by additionally replacing the disability rates of the lower-income group with those of the higher-income group, yielding $f(\mathbf{M}^{H}, \mathbf{D}^{H})$. The disability contribution is

$$\Delta_{\text{disab}} = f(\mathbf{M}^{H}, \mathbf{D}^{H}) - f(\mathbf{M}^{H}, \mathbf{D}^{L})$$

These two components sum exactly to the total gap because

$$\begin{aligned}\Delta_{\text{mort}} + \Delta_{\text{disab}} &= [f(\mathbf{M}^{H}, \mathbf{D}^{L}) - f(\mathbf{M}^{L}, \mathbf{D}^{L})] + [f(\mathbf{M}^{H}, \mathbf{D}^{H}) - f(\mathbf{M}^{H}, \mathbf{D}^{L})] \\ &= f(\mathbf{M}^{H}, \mathbf{D}^{H}) - f(\mathbf{M}^{L}, \mathbf{D}^{L}) = \Delta\end{aligned}$$

The decomposition thus attributes the HALE gap additively to mortality and disability, in that order. By construction, the mortality contribution reflects differences in mortality rates between the two groups, evaluated at the disability level of the lower-income

group; the disability contribution reflects differences in disability rates, evaluated at the mortality level of the higher-income group.

Each aggregate contribution is then further decomposed by age and cause using Horiuchi's method. For the mortality contribution, we define the parameter vector $\boldsymbol{\theta} = (\boldsymbol{\theta}_1, \boldsymbol{\theta}_2)$, where $\boldsymbol{\theta}_1 = \text{vec}(\mathbf{M}^L)$ and $\boldsymbol{\theta}_2 = \text{vec}(\mathbf{M}^H)$ are the vectorized mortality matrices. The contribution of the j-th element (a specific age-cause cell) is given by

$$\delta_j = \int_0^1 \frac{\partial f}{\partial \theta_j}(\boldsymbol{\theta}(t)) \cdot \frac{d\theta_j}{dt} dt$$

where $\boldsymbol{\theta}(t) = \boldsymbol{\theta}_1 + t(\boldsymbol{\theta}_2 - \boldsymbol{\theta}_1)$ for $t \in [0,1]$ is a straight path from the lower-income to the higher-income parameters. In practice, the integral is approximated by dividing $[0,1]$ into N equally spaced intervals and evaluating the partial derivatives numerically. We used $N = n_{\text{age}} \times n_{\text{cause}}$ to ensure sufficient precision. The resulting contributions were reshaped into $K \times C$ matrices for mortality and, analogously, for disability.

Appendix Text S3. Empirical mode decomposition of contribution time series

For each age-cause cell (separately for mortality and disability contributions), we obtained a time series $c_t(t = 2010, \ldots, 2019)$. We decomposed c_t into intrinsic mode functions (IMF) and a residual. The algorithm proceeds by identifying local maxima and minima, constructing upper and lower envelopes via cubic spline interpolation, extracting the mean envelope, and subtracting it from the signal to obtain an IMF. This sifting process is repeated until the residual has at most one extremum. The final residual r_t was taken as the long-term trend. If the series had fewer than five non-missing values or if the empirical mode decomposition (EMD) algorithm failed to converge, we substituted a locally estimated scatterplot smoothing (LOESS) trend with a span of 0.75 as the long-term trend. The trend direction was defined as the sign of the slope from a linear regression of r_t on time. Based on this direction, each series was classified into one of four dynamic archetypes: mortality catch-up (trend < 0), mortality divergence (trend > 0), disability catch-up (trend < 0), or disability divergence (trend > 0).

Appendix Text S4. Clustering of lower-income countries

For each lower-income country, we computed the average cause-age contribution vectors (separately for mortality and disability) over the study period. After concatenating the two vectors, we obtained a feature vector of length $2 \times K \times C$ for each country. We standardized the features to zero mean and unit variance and then performed principal component analysis (PCA) for dimensionality reduction. The first two principal components were retained for visualisation. We used partitioning around medoids (PAM) clustering with Euclidean distance on the full standardized feature space, selecting the optimal number of clusters by maximising the average silhouette width for $k = 2$ to $k = 6$.

Appendix Figure S1. Aggregated long-term trends of the four dynamic archetypes of age-cause contributions to the HALE gap, 2010–2019

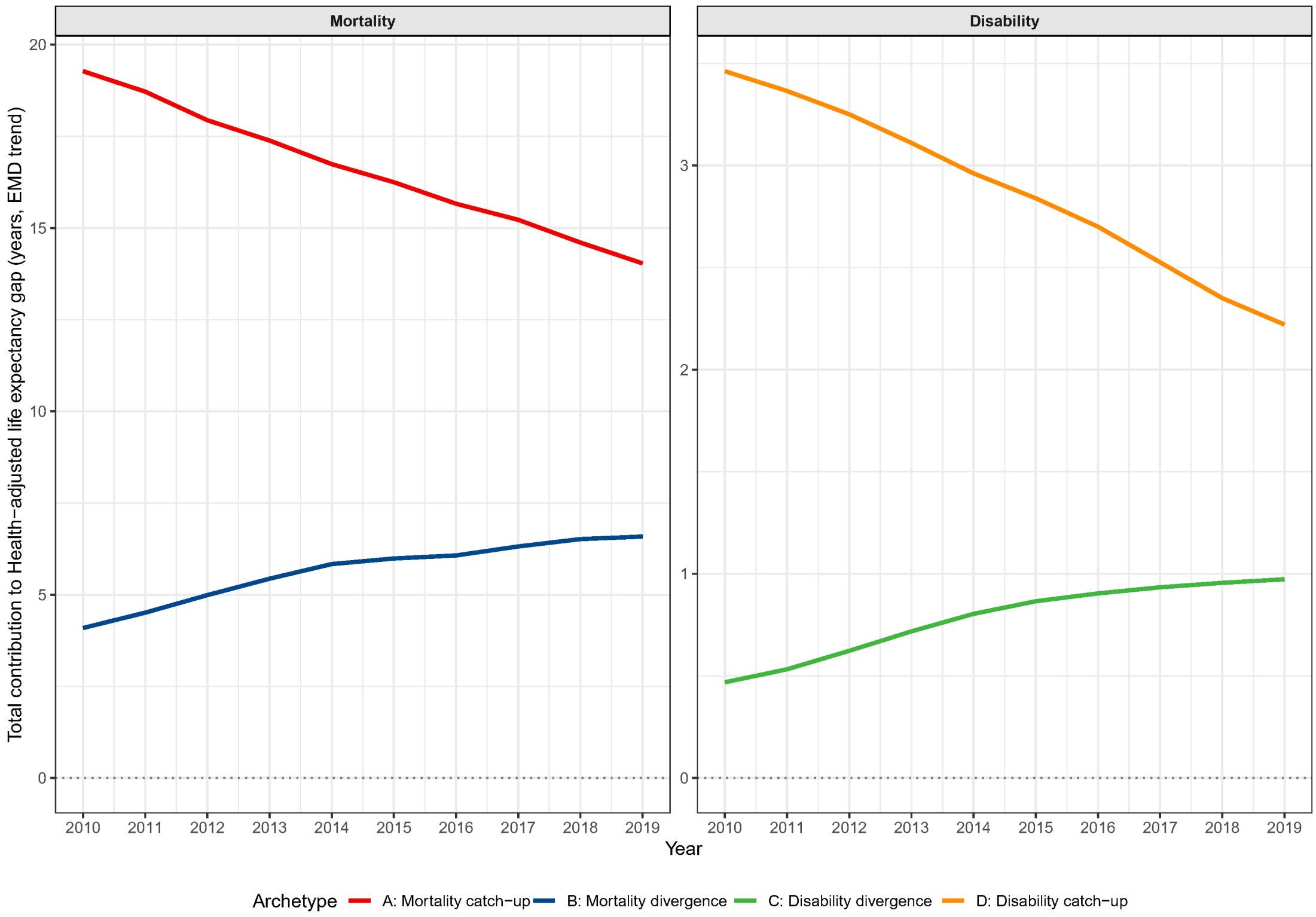

Notes: Each line represents the empirical mode decomposition (EMD) trend of the summed contribution of all age-cause combinations classified into a given dynamic archetype. Archetypes: mortality catch-up (trend narrowing the gap), mortality divergence (trend widening the gap), disability divergence, and disability catch-up. Panels separate mortality and disability contributions. The horizontal dashed line marks zero contribution.

Appendix Figure S2. Mean age-cause contribution profiles for each cluster of lower-income countries, separately for major mortality and disability effects

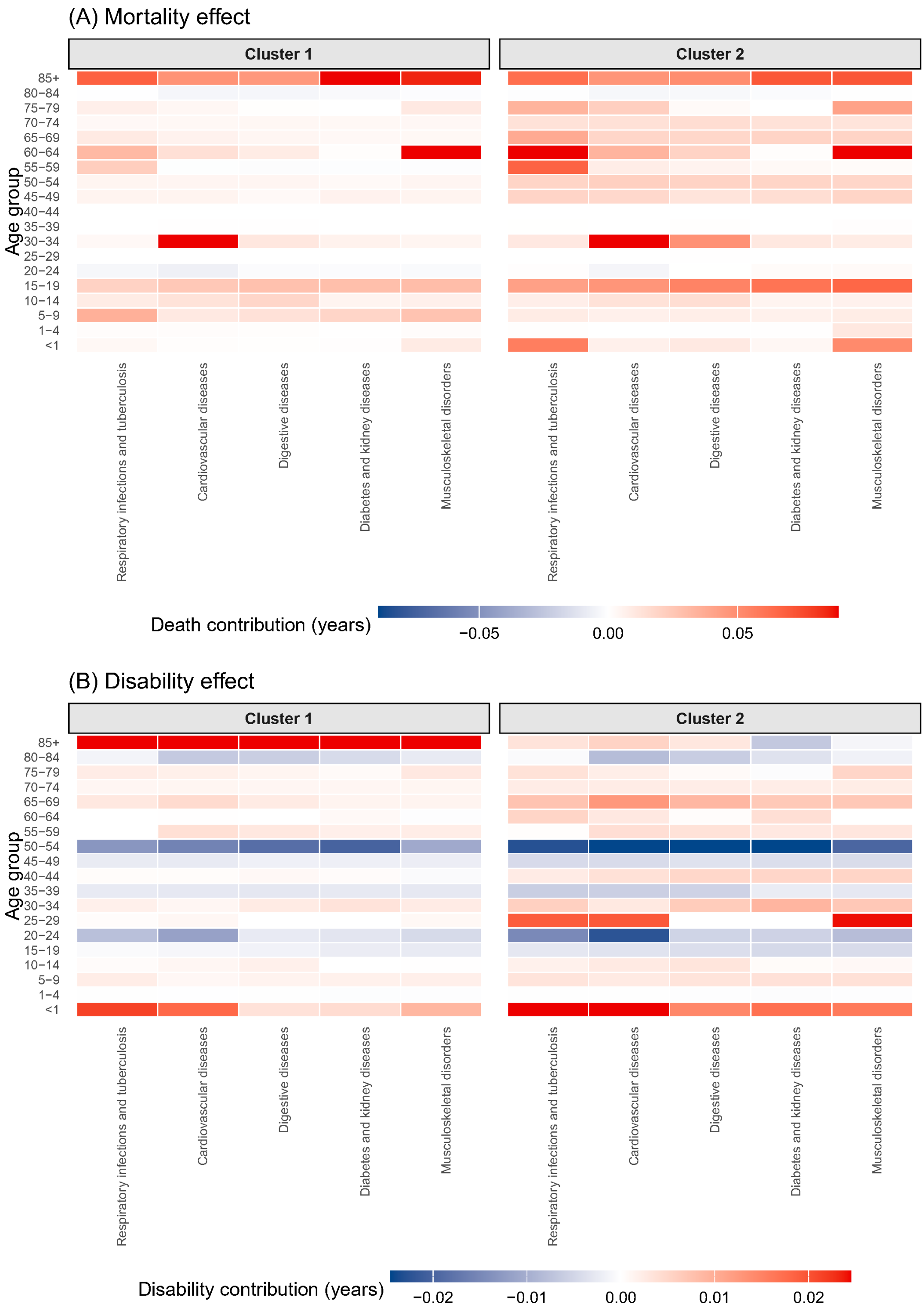

Notes: Heatmaps display the cluster-averaged contribution of each age-cause combination to the HALE gap between higher-income and lower-income countries.

Upper panel: mortality contributions; lower panel: disability contributions. Red cells indicate causes and ages that widen the gap (favouring higher-income countries), while blue cells indicate contributions that narrow the gap.
